# ﻿A new genus and three new species of Hahniidae (Araneae) from China

**DOI:** 10.3897/zookeys.1197.119935

**Published:** 2024-04-18

**Authors:** Lu-Yu Wang, Zhi-Sheng Zhang

**Affiliations:** 1 Key Laboratory of Eco-environments in Three Gorges Reservoir Region (Ministry of Education), School of Life Sciences, Southwest University, Chongqing 400715, China Southwest University Chongqing China

**Keywords:** Comb-tailed spider, description, morphology, taxonomy, Wuling Mountains

## Abstract

A new genus of comb-tailed spider (Hahniidae), *Sinahahnia***gen. nov.**, is described based on three new species from the high-altitude areas of China: *Sinahahniaeyu***sp. nov.** (♂♀, Chongqing and Hubei), *S.fanjingshan***sp. nov.** (♂♀, Guizhou), and *S.yintiaoling***sp. nov.** (♀, Chongqing). Digital images, illustrations, and a distribution map are provided.

## ﻿Introduction

The family Hahniidae is distinguishable from other spider families by its transversely oriented spinnerets. Although it is a widely distributed group, only 28 genera and 236 species have been described globally (WSC 2024). Most hahniid genera have limited distributions, with the exception of the type genus, *Hahnia* C.L. Koch, 1841, which is found throughout Africa, Europe, Asia, and North America.

Recently, several local studies have shown that the diversity of Hahniidae is still not completely explored. For example, Dupérré and Tapia (2024) recently recorded Hahniidae from Ecuador, describing for the first time three new genera and 13 new species. A study on Chinese Hahniidae revealed four new genera and 11 new species from southwest China and adjacent Southeast Asian countries (Chu et al. 2023).

Here, we describe a new genus and three new species of Hahniidae from Wuling Mountains area (Shennongjia in Hubei, Yintiaoling in Chongqing, and Fanjinshan in Guizhou). The most closely related hahniid genus is *Troglohnia* Lin & Li, 2023, which is known from the caves of the Yunnan-Guizhou Plateau, in the western Wuling Mountains.

## ﻿Materials and methods

All specimens are preserved in 75% ethanol and were examined, illustrated, photographed, and measured using a Leica M205A stereomicroscope equipped with a drawing tube, a Leica DFC450 Camera, and LAS v. 4.6 software. Male palps and epigynes were examined and illustrated after they were dissected. Epigynes were cleared by immersing them in pancreatin for about 1 h (Álvarez-Padilla and Hormiga 2007). Eye sizes were measured as the maximum dorsal diameter. Leg measurements are shown as: total length (femur, patella and tibia, metatarsus, tarsus). All measurements are in millimetres. Specimens examined here are deposited in the Collection of Spiders, School of Life Sciences, Southwest University, Chongqing, China (**SWUC**). Terminology follows Zhang and Zhang (2013).

Abbreviations used in the text: **ALE**–anterior lateral eye; **AME**–anterior median eye; **PLE**–posterior lateral eye; **PME**–posterior median eye; **RTA**–retrolateral tibial apophysis.

## ﻿Taxonomy

**Family Hahniidae Bertkau, 1878**

### ﻿ Sinahahnia gen. nov.

Taxon classificationAnimaliaAraneaeHahniidae

9758851D-B8D7-5E00-B5CD-CE64E069084C

https://zoobank.org/28AEBCF1-E818-49C4-9C8B-B4CEAF08C6D2

#### Type species.

*Sinahahniaeyu* sp. nov.

#### Etymology.

The generic name is a compound noun derived from the Latin *sinae* (= the Chinese) and ‘-*Hahnia*’.

#### Diagnosis.

Species of *Sinahahnia* gen. nov. resemble those of *Troglohnia* in having a similar slender embolus, large and membranous median apophysis, short and strong patellar apophysis, and long, spiral copulatory ducts, but they differ by the long embolus originating at a 6-o’clock position (vs 3-o’clock in *Troglohnia*), the large membranous median apophysis originating from the prolateral part of the tegulum (vs retrolateral in *Troglohnia*), RTA not bifurcated (vs bifurcated in *Troglohnia*) (Figs 2A–C, 3C–E, 5A–C, 6C–E cf. Chu et al. 2023: figs 3C, 10A, B, 12A, B, 15A, B), and the peanut-shaped or spherical spermathecae of the epigyne (vs oval in *Troglohnia*) (Figs 2E, 3G, 4C, 5E, 6G, 7B, 8C cf. Chu et al. 2023: figs 11B, 13B, 14B, 16B).

#### Description.

Small size (male: 1.65–1.94, female: 1.39–2.27). Carapace yellowish brown. Eight eyes. Fovea vertical. Cervical groove and radial furrows distinct. Chelicerae, yellowish brown. Labium yellowish brown, wider than long. Endites yellowish brown, longer than wide. Sternum yellowish brown and scutellate with sparse black hairs. Chelicera with 1–3 promarginal and 5 retromarginal teeth. Legs yellowish brown. Leg formula: 4123. Opisthosoma oval, dorsum yellowish brown, dorsally with five light chevrons, venter yellowish brown.

Male palp patella with 1 or 2 apophyses. RTA curved, short, as long as tibia. Cymbial furrow as long as cymbium. Tegulum spherical, about 1/3 of length of cymbium. Median apophysis large and membranous, arc-shaped, originating from the prolateral part of the tegulum. Embolus originating at approximately 6-o’clock position (retrolateral part at 3:30 o’clock), long, slender, curved along bulb, with its tip staying in cymbial furrow near embolic base.

***Epigyne***: epigynal plate wider than long. Copulatory openings small, conspicuous, located mid-ventrally on epigynal plate, not touching each other. Copulatory ducts thin, long, strongly coiled. Secondary spermathecae small, spherical, located anteriorly. Spermathecae peanut-shaped or spherical.

#### Composition.

*Sinahahniaeyu* sp. nov., *S.fanjingshan* sp. nov., and *S.yintiaoling* sp. nov.

#### Distribution.

China (Hubei, Chongqing, Guizhou) (Fig. 9).

### ﻿ Sinahahnia eyu sp. nov.

Taxon classificationAnimaliaAraneaeHahniidae

752589F9-D0BB-527C-931A-AFF49393D16A

https://zoobank.org/ADA60175-515D-4C96-8508-891D21B46501

**Figure 1.**
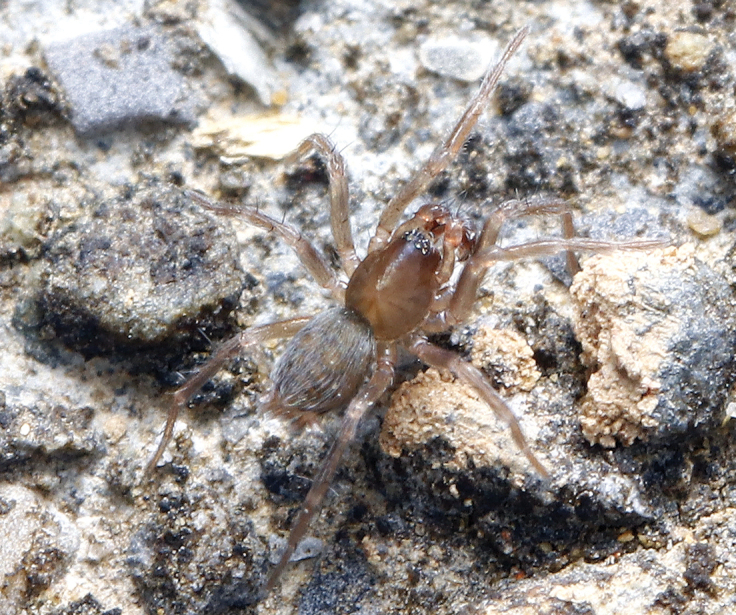
Living male specimen of *Sinahahniaeyu* sp. nov. (photo by Qian-Le Lu).

**Figure 2.**
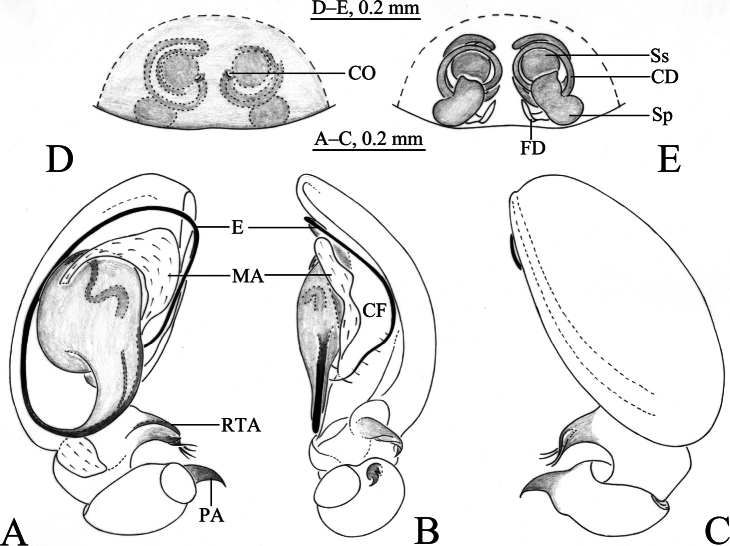
*Sinahahniaeyu* sp. nov. **A–C** holotype male **D, E** paratype female **A** left male palp, ventral view **B** same, retrolateral view **C** same, dorsal view **D** epigyne, ventral view **E** vulva, dorsal view. Abbreviations: CD = copulatory duct; CF = cymbial furrow; CO = copulatory opening; E = embolus; FD = fertilization duct; MA = median apophysis; PA = patellar apophysis; RTA = retrolateral tibial apophysis; Sp = spermatheca; Ss = secondary spermatheca.

**Figure 3.**
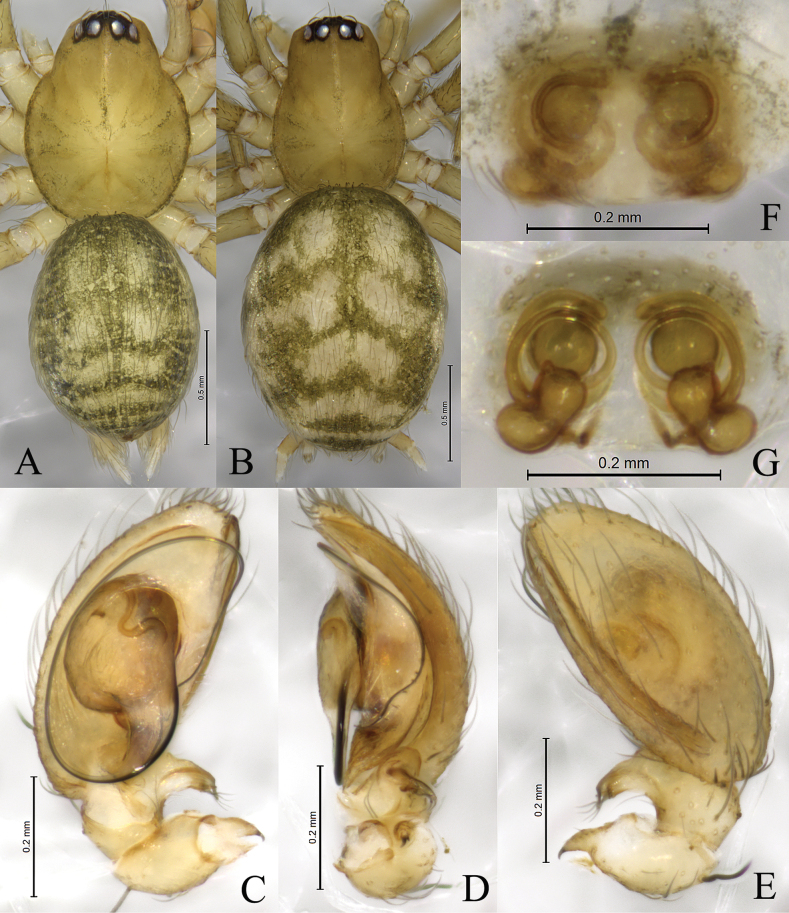
*Sinahahniaeyu* sp. nov. **A, C–E** holotype male **B, F, G** paratype female **A** male habitus, dorsal view **B** female habitus, dorsal view **C** left male palp, ventral view **D** same, retrolateral view **E** same, dorsal view **F** epigyne, ventral view **G** same, dorsal view.

**Figure 4.**
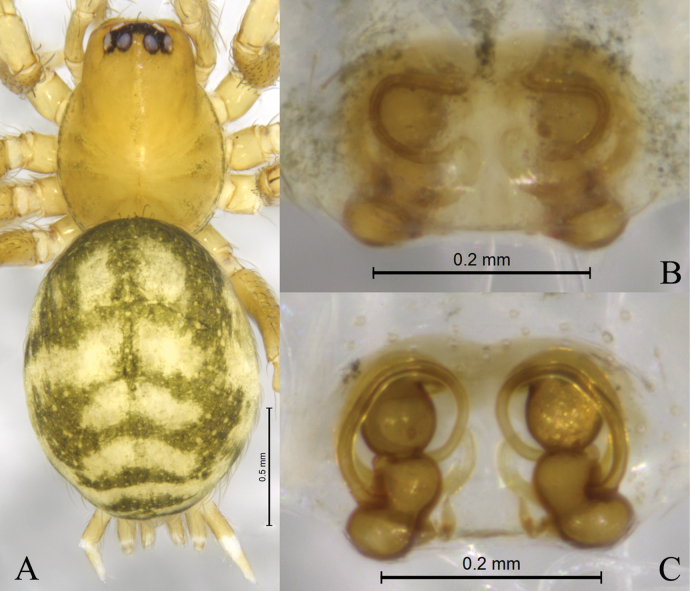
*Sinahahniaeyu* sp. nov., paratype female (SWUC-T-HA-10-07) **A** female habitus, dorsal view **B** epigyne, ventral view **C** same, dorsal view.

Figs 1 , 2 , 3 , 4 , 9

#### Type material.

***Holotype*** ♂ (SWUC-T-HA-10-01), **China**, Hubei Prov., Shiyan City, Zhushan Co., Liulin Township, Duheyuan Nature Reserve, 31°31′50″N, 110°0′29″E, elev. 1678 m, 19 September 2023, L.Y. Wang, et al. leg. ***Paratypes***: 1♂ 4♀(SWUC-T-HA-10-02~06), same data as for holotype • 1♀ (SWUC-T-HA-10-07), Chongqing City, Wuxi Co., Yintiaoling Nature Reserve, Linkouzi, Fenshuihe, 31°29′47″N, 109°55′33″E, elev. 1796 m, 13 April 2022, LY. Wang leg.

#### Etymology.

The specific name is derived from the Chinese word ‘e’ and ‘yu’, E is an abbreviated name for Hubei and Yu is an abbreviated name for Chongqing.

#### Diagnosis.

**Figure 5.**
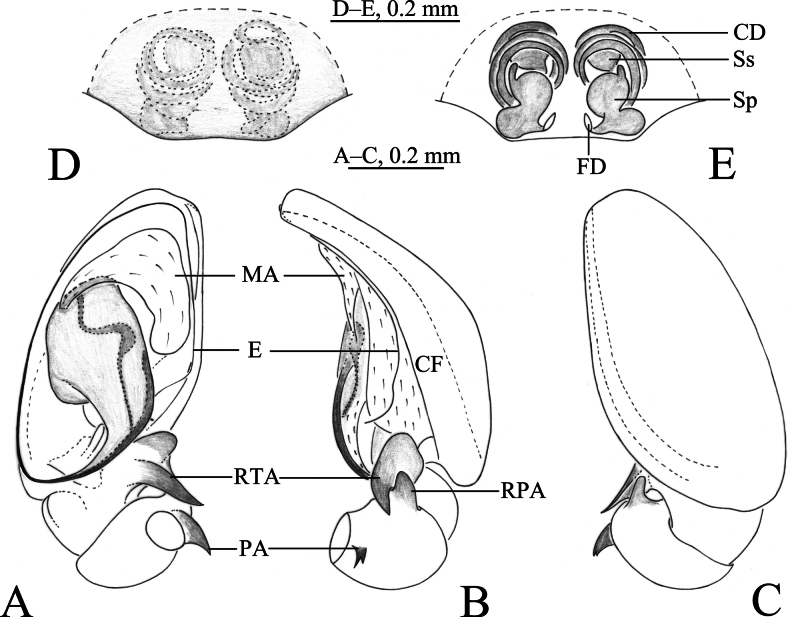
*Sinahahniafanjingshan* sp. nov. **A–C** holotype male **D, E** paratype female **A** left male palp, ventral view **B** same, retrolateral view **C** same, dorsal view **D** epigyne, ventral view **E** vulva, dorsal view. Abbreviations: CD = copulatory duct; CF. = cymbial furrow; CO = copulatory opening; E = embolus; FD = fertilization duct; MA = median apophysis; PA = patellar apophysis; RPA = retrolateral patellar apophysis; RTA = retrolateral tibial apophysis; Sp = spermatheca; Ss = secondary spermatheca.

**Figure 6.**
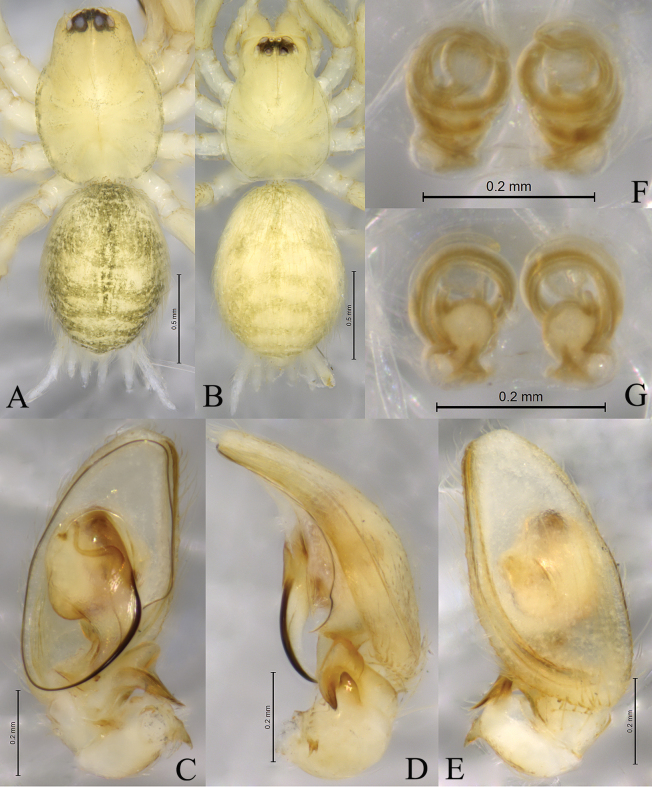
*Sinahahniafanjingshan* sp. nov. **A, C–E** holotype male **B, F, G** paratype female **A** male habitus, dorsal view **B** female habitus, dorsal view **C** left male palp, ventral view **D** same, retrolateral view **E** same, dorsal view **F** epigyne, ventral view **G** same, dorsal view.

The new species resembles *S.fanjingshan* sp. nov. (Figs 5, 6 cf. Figs 2–4) in having a long, slender embolus, a large, membranous median apophysis, and long, spiral copulatory ducts, but the new species differs from the latter by the twisted RTA with small thorns (vs curved and without thorn in *S.fanjingshan* sp. nov.), the single patellar apophysis (vs two in *S.fanjingshan* sp. nov.) (Figs 2A–C, 3C–E cf. Figs 5A–C, 6C–E), the reniform spermathecae (Figs 2E, 3G, 4C cf. Figs 5E, 6G) (vs peanut-shaped in *S.fanjingshan* sp. nov.).

#### Description.

Male holotype (Fig. 3A) total length 1.88. Carapace 0.92 long, 0.73 wide; opisthosoma 0.99 long, 0.76 wide. Eye sizes and interdistances: AME 0.03, ALE 0.06, PME 0.06, PLE 0.07; AME–AME 0.02, AME–ALE 0.01, PME–PME 0.04, PME–PLE 0.02, ALE–PLE 0.02. MOA 0.14 long, anterior width 0.09, posterior width 0.17. Clypeus height 0.09. Chelicerae with 2 promarginal and 5 retromarginal teeth. Leg measurements: I 2.49 (0.70, 0.78, 0.55, 0.46); II 2.29 (0.65, 0.78, 0.49, 0.37); III 1.92 (0.52, 0.56, 0.45, 0.39); IV 2.51 (0.75, 0.75, 0.56, 0.45).

***Palp*** (Figs 2A–C, 3C–E). Patellar apophysis curved, short, about 1/3 length of patella, with sharp tip. RTA spiral, with some small thorns. Cymbial furrow as long as cymbium. Median apophysis membranous. Embolus originating at approximately 6-o’clock position, slender, curved along with bulb, its tip staying in cymbial furrow near embolic base.

One of the paratypes (SWUC-T-HA-10-02, Fig. 3B) total length 2.27. Carapace 0.97 long, 0.70 wide; opisthosoma 1.38 long, 1.08 wide. Eye sizes and interdistances: AME 0.04, ALE 0.08, PME 0.07, PLE, 0.08; AME–AME 0.03, AME–ALE 0.01, PME–PME 0.05, PME–PLE 0.02, ALE–PLE 0.01. MOA 0.15 long, anterior width 0.11, posterior width 0.19. Clypeus height 0.07. Leg measurements: I 2.08 (0.57, 0.71, 0.42, 0.38); II 2.01 (0.60, 0.64, 0.39, 0.38); III 1.76 (0.51, 0.56, 0.36, 0.33); IV 2.33 (0.63, 0.76, 0.52, 0.42).

***Epigyne and vulva*** (Figs 2D–E, 3F, G, 4B, C). Epigynal plate wider than long. Copulatory openings small, located mid-ventrally on epigynal plate. Copulatory ducts thin, long, wrapped three times around secondary spermathecae. Secondary spermathecae small, located anteriorly. Spermathecae reniform, more than twice as large as secondary spermathecae. Fertilization ducts spiral and hook-like.

#### Variation.

Males (*n* = 2) total length 1.76–1.88; females (*n* = 5) total length 1.86–2.27.

#### Distribution.

China (Chongqing, Hubei) (Fig. 9).

### ﻿ Sinahahnia fanjingshan sp. nov.

Taxon classificationAnimaliaAraneaeHahniidae

7115FCF6-1ED0-5EAB-B163-1361476B3415

https://zoobank.org/D59809F3-05ED-4387-813B-8809A9E55739

Figs 5 , 6 , 9

#### Type material.

***Holotype*** ♂ (SWUC-T-HA-11-01), **China**, Guizhou Prov., Tongren City, Songtao Co., Wuluo Town, Fanjingshan Nature Reserve, near Maxi’ao Tunnel, 28°01′09″N, 108°45′24″E, elev. 1239 m, 11 October 2013, L.Y. Wang, D. Wang and X.K. Jiang leg. ***Paratypes***: 1♂ 2♀ (SWUC-T-HA-11-02~04), with same data as for holotype • 12♀ (SWUC-T-HA-11-05~16), Fanjingshan Nature Reserve, Jinding, 27°54′29″N, 108°41′52″E, elev. 2214 m, 29 September 2013, L.Y. Wang, D. Wang and X.K. Jiang leg. • 1♀ (SWUC-T-HA-11-17), Fanjingshan Nature Reserve, Mianxuling, 27°54′32″N, 108°39′49″E, elev. 1974m, 30 September 2013, L.Y. Wang, D. Wang and X.K. Jiang leg. • 1♂ 2♀ (SWUC-T-HA-11-18~20), same data as for holotype except date, 5 October 2013.

#### Etymology.

The specific name is derived from the type locality; it is a noun in apposition.

#### Diagnosis.

The new species resembles *S.eyu* sp. nov. (Figs 2–4 cf. Figs 5, 6) in having a long, slender embolus, a large, membranous median apophysis, and long, spiral copulatory ducts, but the new species differs from the latter in having a curved RTA (vs twisted and with small thorns in *S.eyu* sp. nov.), a bifurcated patellar apophysis (vs single in *S.eyu* sp. nov.), a finger-shaped retrolateral patellar apophysis (vs absent in *S.eyu* sp. nov.) (Figs 5A–C, 6C–E cf. 2A–C, 3C–E), and a peanut-shaped spermathecae (vs reniform in *S.eyu* sp. nov.) (Figs 5E, 6G cf. 2E, 3G, 4C).

#### Description.

Male holotype (Fig. 6A) total length 1.65. Carapace 0.81 long, 0.57 wide; opisthosoma 0.77 long, 0.56 wide. Eye sizes and interdistances: AME 0.02, ALE 0.06, PME 0.05, PLE 0.06; AME–AME 0.02, AME–ALE 0.01, PME–PME 0.04, PME–PLE 0.02, ALE–PLE 0.01. MOA 0.11 long, anterior width 0.04, posterior width 0.16. Clypeus height 0.12. Chelicerae with 1 promarginal and 5 retromarginal teeth. Leg measurements: I 2.19 (0.62, 0.72, 0.46, 0.39); II 1.96 (0.57, 0.62, 0.40, 0.37); III 1.80 (0.52, 0.56, 0.39, 0.33); IV 2.25 (0.63, 0.73, 0.50, 0.39).

***Palp*** (Figs 5A–C, 6C–E). Patellar apophysis curved, short, about 1/3 length of patella, with a bifurcated end. Retrolateral patellar apophysis finger-shaped. RTA curved, as long as tibia. Cymbial furrow as long as cymbium. Median apophysis membranous. Embolus originating at approximately 6-o’clock position, slender, curved along with bulb, its tip staying in the cymbial furrow near embolic base.

One of the paratypes (SWUC-T-HA-11-02, Fig. 6B) total length 1.39. Carapace 0.69 long, 0.50 wide; opisthosoma 0.72 long, 0.53 wide. Eye sizes and interdistances: AME 0.02, ALE 0.04, PME 0.04, PLE, 0.06; AME–AME 0.01, AME–ALE 0.01, PME–PME 0.05, PME–PLE 0.02, ALE–PLE 0.01. MOA 0.09 long, anterior width 0.05, posterior width 0.15. Clypeus height 0.08. Leg measurements: I 1.59 (0.48, 0.51, 0.31, 0.29); II 1.46 (0.45, 0.46, 0.29, 0.26); III 1.33 (0.38, 0.42, 0.28, 0.25); IV 1.72 (0.53, 0.53, 0.37, 0.29).

***Epigyne and vulva*** (Figs 5D, E, 6F, G). Epigynal plate wider than long. Copulatory ducts thin and long, wrapped three times around secondary spermathecae . Secondary spermathecae small, anteriorly located. Spermathecae peanut-shaped, more than twice as large as secondary spermathecae. Fertilization ducts small, hook-like.

#### Variation.

Males (*n*=3) total length 1.65–1.94; females (*n*=17) total length 1.39–1.83.

#### Distribution.

Known only from the type locality in Guizhou, China (Fig. 9).

### ﻿ Sinahahnia yintiaoling sp. nov.

Taxon classificationAnimaliaAraneaeHahniidae

F46CD9AD-E978-5A85-887D-938C28A09DFB

https://zoobank.org/663B4C66-F4DB-474D-8D6A-E20DEEE56931

Figs 7 , 8 , 9

#### Type material.

***Holotype*** ♀ (SWUC-T-HA-12-01), **China**, Chongqing City, Wuxi Co., Yintiaoling Nature Reserve, Guanshan, Shizhuzi, 31°32′15″N, 109°41′49″E, elev. 2147 m, 1 September 2020, Z.S. Zhang, L.Y. Wang, Y. Zhang and P. Liu leg. ***Paratypes***: 8♀ (SWUC-T-HA-12-02~09), with same data as for holotype.

#### Etymology.

The specific name is derived from the type locality; it is a noun in apposition.

#### Diagnosis.

**Figure 7.**
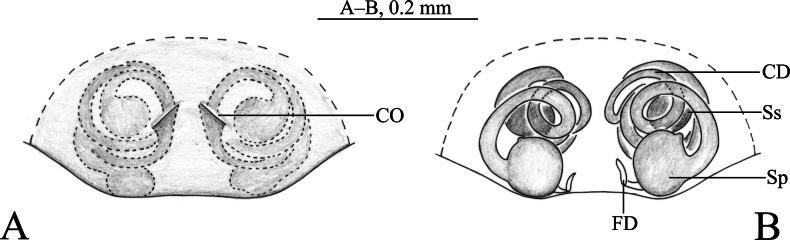
*Sinahahniayintiaoling* sp. nov., holotype female **A** epigyne, ventral view **B** vulva, dorsal view. Abbreviations: CD = copulatory duct; CO = copulatory opening; FD = fertilization duct; Sp = spermatheca; Ss = secondary spermatheca.

**Figure 8.**
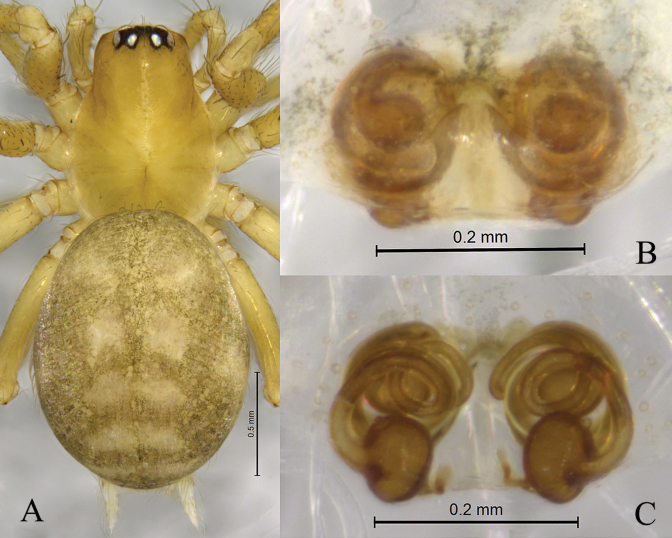
*Sinahahniayintiaoling* sp. nov., holotype female **A** female habitus, dorsal view **C** epigyne, ventral view **D** same, dorsal view.

The new species resembles *S.eyu* sp. nov. (Figs 2–4 cf. Figs 7, 8) in having long and spiral copulatory ducts, but the new species differs from the latter in the large copulatory openings and the spherical spermathecae (vs small copulatory openings and reniform spermathecae in *S.eyu* sp. nov.) (Figs 7, 8B, C cf. Figs 2D–E, 3F, G, 4B, C).

#### Description.

Female holotype (Fig. 8A) total length 2.23. Carapace 1.01 long, 0.74 wide; opisthosoma 1.35 long, 1.04 wide. Eye sizes and interdistances: AME 0.03, ALE 0.06, PME 0.07, PLE 0.08; AME–AME 0.02, AME–ALE 0.01, PME–PME 0.06, PME–PLE 0.03, ALE–PLE 0.01. MOA 0.17 long, anterior width 0.10, posterior width 0.18. Clypeus height 0.09. Chelicerae with 3 promarginal and 5 retromarginal teeth. Leg measurements: I 2.23 (0.66, 0.75, 0.43, 0.39); II 2.09 (0.65, 0.65, 0.42, 0.37); III 2.00 (0.58, 0.62, 0.41, 0.39); IV 2.51 (0.72, 0.81, 0.57, 0.41).

***Epigyne and vulva*** (Figs 7A, B, 8B, C). Epigynal plate wider than long. Copulatory openings small, located mid-ventrally on epigynal plate. Copulatory ducts thin, long, wrapped four times around secondary spermathecae. Secondary spermathecae small, anteriorly located. Spermathecae spherical, more than twice as large as secondary spermathecae. Fertilization ducts small, hook-like.

Male unknown.

#### Variation.

Females (*n*=9) total length 2.06–2.23.

#### Distribution.

**Figure 9.**
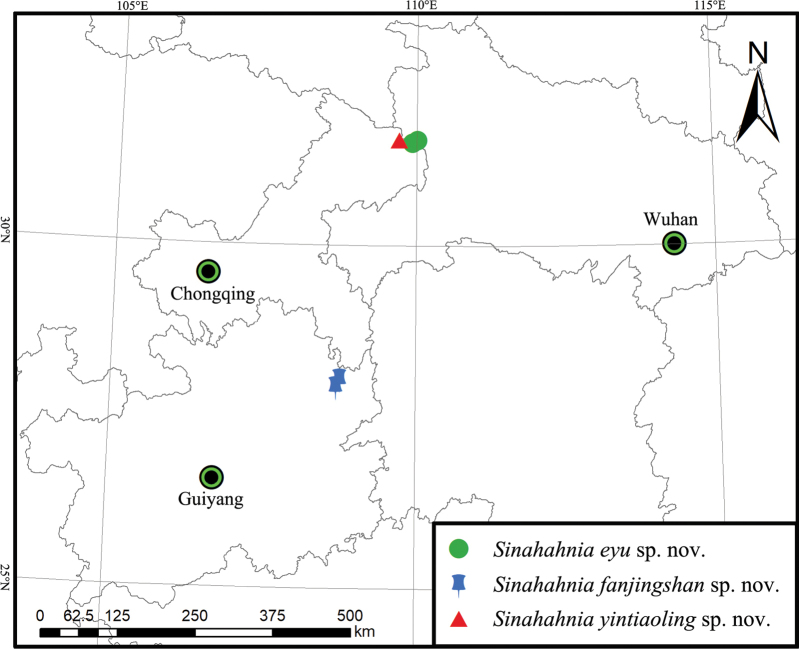
Distribution of *Sinahahnia* in China.

Known only from the type locality in Chongqing, China (Fig. 9).

## Supplementary Material

XML Treatment for Sinahahnia

XML Treatment for Sinahahnia eyu

XML Treatment for Sinahahnia fanjingshan

XML Treatment for Sinahahnia yintiaoling

## ﻿Additional information

### Author ORCIDs

Lu-Yu Wang https://orcid.org/0000-0002-5250-3473

Zhi-Sheng Zhang https://orcid.org/0000-0002-9304-1789

### Data availability

All of the data that support the findings of this study are available in the main text.
